# The brain is not mental! coupling neuronal and immune cellular processing in human organisms

**DOI:** 10.3389/fnint.2023.1057622

**Published:** 2023-05-17

**Authors:** Anna Ciaunica, Evgeniya V. Shmeleva, Michael Levin

**Affiliations:** ^1^Centre for Philosophy of Science, Faculty of Science, University of Lisbon, Lisbon, Portugal; ^2^Faculty of Brain Sciences, Institute of Cognitive Neuroscience, University College London, London, United Kingdom; ^3^Department of Biology, Tufts University, Medford, MA, United States; ^4^Allen Discovery Center, Tufts University, Medford, MA, United States

**Keywords:** neural system, immune system, self-organization, cellular systems, embodiment

## Abstract

Significant efforts have been made in the past decades to understand how mental and cognitive processes are underpinned by neural mechanisms in the brain. This paper argues that a promising way forward in understanding the nature of human cognition is to zoom out from the prevailing picture focusing on its neural basis. It considers instead how neurons work in tandem with other type of cells (e.g., immune) to subserve biological self-organization and adaptive behavior of the human organism as a whole. We focus specifically on the immune cellular processing as key actor in complementing neuronal processing in achieving successful self-organization and adaptation of the human body in an ever-changing environment. We overview theoretical work and empirical evidence on “basal cognition” challenging the idea that only the neuronal cells in the brain have the exclusive ability to “learn” or “cognize.” The focus on cellular rather than neural, brain processing underscores the idea that flexible responses to fluctuations in the environment require a carefully crafted orchestration of multiple cellular and bodily systems at multiple organizational levels of the biological organism. Hence cognition can be seen as a multiscale web of dynamic information processing distributed across a vast array of complex cellular (e.g., neuronal, immune, and others) and network systems, operating across the entire body, and not just in the brain. Ultimately, this paper builds up toward the radical claim that cognition should not be confined to one system alone, namely, the neural system in the brain, no matter how sophisticated the latter notoriously is.

## 1. Introduction

The idea that the mind is distinct from the body and somehow at home in the human brain has deep roots in a longstanding philosophical and scientific thinking, stretching from Antiquity to the present day (Bennett and Hacker, 2003). At least two underlying and intertwined assumptions guided heated debates around the mind and body distinction in the past centuries. First there is the assumption that “inner” mental psychological states such as pain are distinct from physical matter. Second, there is the idea that there is a theoretical problem of how humans can know or cognize the “external” physical world. Starting mid- 1960s, the view that humans and other psychological organisms are best viewed as information-processing systems cognizing the world became dominant (Fodor, 1968). This view accepted the idea that one must appeal to “inner” states (e.g., pain) to explain ‘visible’ external behavior (e.g., crying), provided the former are construed as physical states. Mental states thus are not ghostly or non-physical, but rather neurophysiological events occurring in the individual’s nervous system (Smart, 1959).

Cognition thus was mainly defined as a rule-governed manipulation of mental representations in the brain, following the example of digital computers. The study of mental and cognitive states and the brain became thus intertwined, as exemplified by a plethora of handbooks and popular introductions on mind and cognition (Lycan, 1990; Braddon-Mitchell and Jackson, 1996). Broadly speaking, the field of cognitive psychology organized its research agenda around the key question: “how does this organism receive information through its sense-organs, process the information, store it, and the mobilize it in such a way as to result in intelligent behavior?” (Lycan, 1990: 8). Similarly, the field of cognitive neuroscience focuses on how the brain receives and process the information in a such a way to result in adaptive behavior.

The embodied cognition paradigm has been heralded as an alternative to the mainstream cognitive science (Varela et al., 1991). A central tenet of this approach is that the mechanisms of cognition have evolved to assist biological organisms in their adaptive interactions with the environment (Maturana and Varela, 1980). Cognition is thus first and foremost a process of information processing geared to sustain and maintain the physico-, biochemical-, and bioelectrical processes that constitute a biological self-organizing organism: the human body (Thompson, 2007; Lyon, 2020; Levin, 2021a,b). Here we define cognition minimally as information processing within a self-organizing system.

The notion of self-organization was seminally introduced in the field of cybernetics (Ashby, 1947; Foerster, 1960) and expanded subsequently to various disciplines including physics, biology (Camazine, 2003) and neuroscience (Kelso, 1997; Friston, 2010; Tognoli and Kelso, 2014). Self-organization is typically defined as the spontaneous emergence of spatio-temporal order or pattern-formation processes in both physical and biological systems resulting from interactions of its components with the environment (Camazine et al., 2001; Rosas et al., 2018).

Biological self-organization is a notion extensively used in theoretical biology to refer to processes and mechanisms allowing biological systems to resist the natural tendency to disorder. The special case of biological systems in the natural world is nicely captured in an aphorism by La Cerra and Bingham reported by Lyon (2020): “the first law of psychology is the second law of thermodynamics.” The second law stipulates that closed physical processes tend toward a state of increasing statistical probability and decreasing order, leading ultimately to a thermodynamic equilibrium (Schr’´odinger, 1944).

Recent work has described the remarkable capacity of biological organisms such as human bodies to maintain themselves in a state far from thermodynamic equilibrium (Von Bertalanffy, 1968; Friston et al., 2017) by building upon the Free Energy Principle (FEP) (Friston, 2010). FEP is a formalization and extension of the Schr’´odinger (1944) seminal idea that living organisms avoid entropy, by engaging in self-organization with the goal of maintaining their internal states within optimal limits for survival (Maturana and Varela, 1980). The continuous process of minimizing free energy allows biological systems to avoid increase of entropy and hence dissipation and decay (Clark, 2013; Hohwy, 2014; Perunov et al., 2014; Chvykov et al., 2021).

Biological self-organization encompasses the emergence of coherent structural configurations and patterns that distinguish microscopic (e.g., cells) and macroscopic (e.g., organisms) systems from their environment (Sultan, 2015; Sultan et al., 2022). Specifically, it has been proposed that the spatial confinement enables cells to function in a form of self-organizing chemical activity patterns (Turing, 1952), and to control the flow of matter and energy in order to maintain themselves in entropy-dissipating non-equilibrium conditions (Schr’´odinger, 1944; Prigogine and Nicolis, 1967). A richly detailed body of evidence illustrated that biological organisms such as complex bodies need to develop and sustain robust yet flexible self-organizing and self-regulatory mechanisms implemented via multilevel hierarchical organization: organelles constitute cells which form tissues which in turn form organs, etc., Zeng (2022).

The dynamics of biological systems include complex phenomena such as chaos, bifurcation, patterning, dissipation, and synchronization (Kapitaniak and Jafari, 2018). It also includes context –responsiveness to developmental, metabolic, immune and endocrine processes. In their seminal work, the biologists Maturana and Varela (1987) proposed the notion of “autopoiesis” to describe the minimal self-organization of living systems, focusing on the metabolic self-production of single-cell organisms and homeostatic regulation. Homeostasis is defined as “the regulation by an organism of the chemical composition of its body fluids and other aspects of its internal environment so that physiological processes can proceed at optimum rates. It involves monitoring changes in the external and internal environments by means of receptors and adjusting the composition of the body fluids accordingly; excretion and (osmotic) regulation are important in this process” (Martin and Hine, 2000). Biological systems also expand on this basic scheme to implement allostasis (McEwen, 1998; Schulkin and Sterling, 2019), and homeorhesis (Colditz, 2020; Matsushita and Kaneko, 2020).

Which enable the organism to take a more active role in dealing with its environment and its own components.

Self-organizing autonomous systems are organizationally closed such that the network of processes is recursively dependent on each other in the generation and realization of the processes themselves (Rosen, 2005). Moreover, they constitute the system as “unity recognizable in the space (domain) in which the processes exist” (Varela, 1979:55). Self-organization in living systems must feature the emergence of boundaries that define an internal space – the boundaries of the Self (Levin, 2021a), while keeping the states coupled with their surroundings (Palacios et al., 2020). Dynamical and precarious systems endowed with open boundaries may be seen as a self-organizing system striving to maintain its functional and structural integrity.

In line with the embodied cognition view (Maturana and Varela, 1980), it has been suggested that a promising way forward is to regard the “principles of biological organization and the requirement of survival and reproduction present the most productive route to a general understanding of the principle of cognition” (Lyon, 2006:12). A corollary of this approach is to define cognition as “the sensory and other information-processing mechanisms an organism has for becoming familiar with, valuing, and interacting productively with features of its environment (exploring, exploiting, and evading) in order to meet existential needs, the most basic of which are survival/persistence, growth, thriving, and reproduction” (Lyon, 2006: 416).

It is generally accepted that the body and the brain are distinct and partially independent subsystems working in tandem to ensure the organism’s survival in an ever-changing environment. It is also established that the human brain actively participates in this vital task by sustaining and maintaining optimal and flexible neurophysiological and cognitive processing subserving bodily integrity and adaptive worldly interactions. It is less understood however, how cognitive processing emerge from neural processing. Despite significant combined efforts from neurobiology and neuroscience using increasingly sophisticated tools such brain imaging, genetic manipulation and fluorescent labeling (Dennett, 1992; Damasio, 2000; Seth and Tsakiris, 2018), the question how exactly brain (i.e., neural) activity generates cognitive and mental states remains fairly open.

In this paper, we suggest that one promising way forward in addressing this key question is to zoom out from the prevailing focus on the neural/mental states relationship and to consider instead how neurons (i.e., a certain type of cells) work in tandem with other type of cells (e.g., immune) to subserve self-organization and adaptive behavior of the human organism as a whole.

We build upon the key fact that the brain *is* (part of) the body and as such, like any other bodily organ, the brain is made of cells. The focus on cellular rather than neural, brain processing allows us to underscore the idea that flexible responses to changes in the environments requires flexible adjustments not only through neural, but also through metabolic, cellular and immunological processing at multiple organizational levels of the biological system. We focus specifically on the immune system processing as key actor in complementing brain systems processing to achieve successful self-organization and adaptation of the human organism. Ultimately, shifting the focus from neural to cellular processing invites us to reconsider the received idea that cognitive processes can be linked solely to the neural system, and that the brain is somehow the natural home of mental states.

In (section “1. Introduction”), we motivate the shift in focus from neural to cellular processing and briefly describe their fundamental role of the latter in constituting biological self-organizing systems such as the human body. In (section “2. Cells: the fundamental units of the brain and body”), we discuss existing body of work on “basal cognition” (Baluška and Levin, 2016; Levin, 2019, 2021a,b; Lyon et al., 2021) questioning the prevailing idea that only brains (i.e., collectives of neuronal cells) has the ability to “cognize” or “learn.” This discussion motivates the idea that non-neural cells and simple organisms may also be perceived as active primitive “cognizers.” (section “3. Cells as “smart cognizers?” The simple minds-complex life continuity thesis”) introduces the idea that being fundamentally a *bodily* system, the brain needs to carefully orchestrate and align its neural processing with a complex network of other types of cellular processing (e.g., immune) to ensure the organism’s survival and viable interactions with the world. The focus on the immune system complementing the neural system to jointly support the cognitive processing underlying self-organization of the human body. In (section “4. Coupling neuronal and immune processing in human embodiment”) we suggest that cognitive processes are better understood as multiscale processes implemented at multilevel bodily systems and intricate cellular networks that compose the biological human organism as a whole.

## 2. Cells: the fundamental units of the brain and body

A central tenet of prevailing approaches in brain sciences is the intrinsic link between brain (i.e., *neural*) processing and *cognitive* processing, as illustrated by a the name of the field: cognitive neuroscience. While neurons are fascinating indeed and rightfully placed under exploratory spotlight, here we focus on the more general class of agential materials from which bodies are made: cells.

Neurons are one particular type of cells - the basic, fundamental units of living organisms such as the human body (Mazzarello, 1999). Importantly, it has been proposed that “understanding the organization and function of cells within an organisms lays the essential foundation for understanding how an organism works” (Zeng, 2022: 2739), and it has been suggested that the remarkable capabilities of brains reflect an evolutionary pivot across problem spaces. Neurons are speed-optimized versions of cells that enable them to shift from their solving problems in various spaces, such as anatomical morphospace, by using developmental bioelectricity, to solving behavioral problems in 3D space via rapid control of muscle motion (Fields and Levin, 2022).

Studies going back to Ramon y Cajal revealed that cell types in the brain and body display several properties in many modalities (e.g., molecular, morphological, physiological, and functional) (Zeng and Sanes, 2017; see Zeng, 2022 for a recent review). Cellular identity as defined through morphology and function is a product of intracellular signaling networks that communicate between cells (Koseska and Bastiaens, 2017).

Large organisms such as humans have billions to trillions of cells in the body (Regev et al., 2017; Armand et al., 2021). Community efforts have been made to create cell types atlases for all organs of the human body and the brain). The separation of the neuronal and non-neuronal cells classes constitutes a fundamental distinction [see Zeng (2022): 2743]. The basic architecture of the mammalian brain (Swanson, 2000, 2012) is composed of telencephalon, diencephalon mesencephalon (midbrain), and rhombencephalon (hindbrain). Within each of these major brain structures, there are multiple regions and subregions, each with many cell types. A cell type can be specific to a subregion, a region, or a major brain structure. In each of these areas, there are two neuronal classes based on the dominant neurotransmitters they release, namely, glutamatergic and GABAergic, as well as several non-neuronal classes. A significant body of work on the mammalian brain (typically mice) have revealed a hierarchical organization (Brain Initiative Cell Census Network [BICCN], 2021). These billions to trillions of cells need to carefully orchestrate their exchanges with each other and with the external environment to allow the organism to successfully survive and grow.

It is generally held that cognition requires a nervous system, without which no mental states can arise. Neuronal activities in brain circuits generate sensory perception, cognition and ultimately behavior. For example, it has been proposed that from an evolutionary and ontogenetic perspectives, at the lowest level of the mind are processes anchored in homeostatic neural processing (Damasio and Carvalho, 2013). Feelings, i.e., mental experiences of body states, “are rooted in events occurring at single-cell level, specifically in the unmyelinated axons conveying signals from humoral and visceral aspects of the body toward nuclei in the CNS.” (Damasio and Carvalho, 2013:143) Hence, the deep roots of sophisticated mental and cognitive processing may be traced back to the humble origins of information processing in the metabolic homeostatic mechanisms of ancient cells (Damasio and Carvalho, 2013).

Conversely, bodily states and movements may directly influence neural spiking and oscillatory activity–modulating information processing, perception, action, cognition, and emotion regulation (Critchley and Garfinkel, 2018). A commonly accepted model is that synaptic firing at the single-neuron level is amplified via temporal synchronization, into a system level phenomenon (Engel et al., 1999; Singer, 1999). In biological systems, system-level properties such as fear or boredom, say, are often highly emergent, with gene-regulatory or bioelectric circuits dynamics linking initial state information and transformation rules to large scale structure and function.

For developing biological self-organizing systems, “novelty and stability are the two sides of the same coin” (Sultan et al., 2022: 5). To attain and maintain stable end states in order to reliably secure the organism stability, cellular and neuronal processing need to constantly and flexibly adjust in a context-dependent manner. Even the most direct level of gene expressions is shaped by conditions within and outside the cells, revealing the importance of plasticity, adaptive responsiveness and developmental flexibility in maintaining biological self-organization of the system. Indeed, “processes mediated by the parent’s encounter with its environment may influence the development, physiology and behavior of its offspring, ecologically important ways” (Sultan et al., 2022:3).

An extensive discussion on how different type of cellular (neuronal and non-neuronal) mechanisms develop and function as responsive and adaptive systems influencing and integrating the effects of their cognitive, bodily and environmental processes, lies beyond the scope of this paper. In what follows, we briefly overview existing work addressing basic mechanisms and processing that can be characterized as “basal cognition” in simple organisms (Lyon et al., 2021). The aim is to provide potential evidence building toward the idea that neuronal processes in the brain may not hold a monopoly on cognition. Indeed, non-neural cells may have a causal effect on neural cells. The radical claim is that all cells are cognitive, in the relevant functional sense, and consequently many other body subsystems are cognitive (not just the neuronal one).^1^

## 3. Cells as “smart cognizers?” The simple minds-complex life continuity thesis

Recently, several theorists leveraged a growing body of evidence from neurobiology and biochemistry to suggest that cognitive categories such as “sensing,” “memory” and “learning” can be applied non-metaphorically to the behavior of simple organisms such as bacteria (Lyon, 2015; Prindle et al., 2015; Lyon et al., 2021; see Koseska and Bastiaens, 2017 for a review). This approach echoes the so-called “biogenic explanations of cognition” (Lyon, 2006) which starts with the facts of biology as the basis for theorizing and works “up” to the human case by asking psychological questions as if they were biological questions. This approach is to be contrasted with the “anthropogenic” approach which assumes human cognition as the paradigm and works “down” to a more general explanatory concept. The key assumption underlying the biogenic approach is that the “information-processing dynamics of “simpler” forms of life are part of a continuum with human cognition” (Lyon et al., 2021:2; see also Baluška and Levin, 2016).

Importantly, as Lyon et al. (2021) note, “the molecular infrastructure for capacities typically associated with brains long predated the evolution of neurons” (2). For example, Liu et al. (2015) observed a close relationship between electrical signaling in bacterial biofilms and information-processing in mammalian brains, proposing a “parallel between neurons and bacteria” related to encoding memory by changes in the membrane potential [echoing prior work on electrical excitability in bacteria (Kralj et al., 2011) and cancer cells]. As Lyon et al. (2021) note, even more remarkable was the finding that more than 95% of these nervous system related genes, including some involved in neural and brain morphogenesis, were commonly shared with *Drosophila, C. elegans*, and *Homo sapiens*: “(approximately)” 30% of planarian system-related genes had homologous sequences in the (plant) Arabidopsis and yeast, which do not possess a nervous system. This implies that the origin of the nervous system-related genes greatly predated the emergence of the nervous system, and that these genes might have been recruited toward the nervous system.” (Mineta et al., 2003: 7666).

More recently, one of us (ML) proposed that “regenerative biology and controlled chimerism reveal that studies of cognition in intact, “standard” evolved animal bodies are just a narrow slice of a much bigger and as-yet unexplored reality: the incredible plasticity of dynamic morphogenesis of biological forms that house and support diverse types of cognition” (Levin, 2019:1). Many fascinating examples from experimental biology illustrate that the boundaries separating somatic and cognitive Selves are fluid. Specifically, we have argued that developmental (pre-neural) bioelectricity sheds new light on how the dynamic control of growths and form of the body evolved into complex cognitive abilities (Levin, 2021a,b). This view holds also for synthetic biology and offers support in favor of a “bacterial paradigm for memory-capable biological systems” (Yang et al., 2020).

Taken together, these empirical findings and theoretical work seem to support the idea that basal cognition may not require nervous system or brain (Levin, 2019). This is because many organisms, including aneural ones display proto-cognitive functions such as memory, prediction and learning. In addition, many aneural organisms show the capacity to flexibly adapt and learn in new contexts (Balázsi et al., 2011). Hence, in order to understand how the physiological activity in individual cells and organisms leads to coherent behavior, one needs to widen the cognitive landscape in order to include aneural organisms, somatic organs, and novel bioengineered synthetic forms among basic cognitive systems (Baluška and Levin, 2016; Levin, 2019; Lyon et al., 2021). Crucially, all of these basal organisms (and individual cells) routinely solve problems (illustrating varying degrees of learning and other capacities) in a variety of unconventional spaces, such as metabolic, transcriptional, physiological, and anatomical (Fields and Levin, 2022) @. Moreover, cognitive abilities are typically taken to be “the properties of a fixed, embodied Agent; the fact that it is a collection of cells or subcellular fragments, which proliferate and actively interact to build up its body, is relegated to developmental biologists. That phase of a subject’s life is usually ignored as behind-the-scenes setup, after which real study can begin” (Levin, 2019:2). Thus, it is essential to broaden consideration of key issues in the field of cognitive science to include the evolutionary and developmental stages (i.e., unconventional embodiments) that smoothly and continuously lead to the appearance of individual organisms. That is, after the embodied turn, cognitive science may need a developmental turn.

Now, as we saw earlier in (section “1. Introduction”), the architecture of the human brain supports a remarkable amount of communication and integration of neural cell signaling. This complex signaling is however, directed at and connected to other non-neuronal cells and network systems subserving the viable functioning of the self-organizing organism as a whole. Indeed, neurons need not only to “fire and wire” together. They also need to wire with and respond to a vast array of other type of cellular networks and bodily organs to produce flexible responses and behavior. Biochemical components and exchanges between neuronal and non-neuronal cells networks constantly interact in influencing downstream processes in multiple systems (cardiac, respiratory, endocrine, immune, gastric, etc.). Empirical evidence illustrates bidirectional influences between bodily physiological signals (e.g., such as heart activity, gastric), brain function and behavior (Park and Tallon-Baudry, 2014; Park and Blanke, 2019; Bhat et al., 2021; Criscuolo et al., 2022; Grund et al., 2022).

We next discuss the idea that neuronal processing in self-organizing biological systems such as the human body is intricately and dynamically linked to non-neuronal processing. Given space limitations, we restrict focus on the immune system only, but the same claim arguably holds for other non-neuronal cellular networks constituting the human body.

## 4. Coupling neuronal and immune processing in human embodiment

All cells of the organism originate from a single cell, the zygote, are closely interconnected, mutually influence one another, and operate in synchrony to achieve common goals–maintenance of homeostasis and constant adaptation to changes of the surrounding environment. In what follows, we depict cognitive processes carried out by non-neural cells, with a particular emphasis on the immune system.

Traditionally, the immune system is described as comprising two parts: the innate and adaptive immune systems. The innate immune system serves as the first line of defense, providing a rapid, non-specific response to pathogens, while the adaptive immune system can be trained to recognize and target specific antigens. However, this classification no longer fully encompasses our current understanding of the immune system. The immune system is a cellular network capable of distinguishing between self, non-self, missing-self, and aberrant-self, including misplaced cells and aberrant intracellular and extracellular molecules (Takeuchi and Akira, 2010; Coers, 2013; Iwasaki and Medzhitov, 2015; Di Virgilio et al., 2020; Zindel and Kubes, 2020). Functions of the immune system include detection, recognition, and elimination of pathogens, foreign substances, cancer cells, or damaged cells. It also plays a key role in inflammation, tissue repair, tissue remodeling, and regulation of immune response magnitude. A properly functioning immune system maintains a balance between responding to harmful and tolerating harmless agents or, in some cases, even tolerating harmful agents (Medzhitov, 2008; Medzhitov et al., 2012). In addition to classical immune functions, the immune system also regulates the nervous system, behavior, metabolism, thermogenesis, and participates in the fight-or-flight response (Dantzer et al., 2008; Rankin and Artis, 2018; Medzhitov, 2021).

The immune network encompasses dozens of distinct immune cell subsets, which communicate with each other and other cells through various means, including cytokines (interleukins, interferons, tumor necrosis factors), chemokines (chemotactic cytokines), various receptors, cell-to-cell interactions (immunological synapses, gap junctions), exosomes and macrovesicles, the complement system, hormones, and neuronal signaling. Along with the ability to interact at a distance by utilizing different molecules, immune cells are motile and can enter and exit the vascular system. As a result, they can migrate across various tissues and organs, which facilitates the coordination of immune processes and immune functions throughout the entire body. A key aspect of this system is the integration of diverse physiological information across distance in the organism, toward an adaptive response in a variety of changing conditions. Consistent with its role as an example of basal cognition, the immune system has been modeled as performing pattern recognition and classification (Carter, 2000).

The human immune system is thus composed of a complex network of numerous specialized cells distributed across the body (Rieckmann et al., 2017; Shilts et al., 2022). Complex arrays and maps of cell-surface proteins coordinate immune cells into inter-connected hubs, linking individual cells through physical interactions (Bergthaler and Menche, 2017). Remarkably, despite being composed of highly dynamic cell types constantly migrating throughout the body, the immune system is designed to flexibly organize its intercellular connections to respond to potential threats to the whole organism.

Importantly, the functional anatomy of the immune system is a key factor in the immune response. The body configuration matters as well as the spatial encapsulation of cells, necessary to spatially organize their chemical components in a such a way that time and place of molecular interactions are a necessary element of their effect (Farnsworth et al., 2013). These interactions are key for both intercellular signal communication and structural cohesion, holding literally the network together, and enabling successful tracking of self-not-self processing (Bausch-Fluck et al., 2018).

It has been proposed that the functions of the immune system are more complex than commonly thought, going beyond the self-not-self discrimination (Cohen and Efroni, 2019). In addition to shielding the body from pathogen, the immune network welcomes and orchestrates complex inflammatory responses designed to sustain the body and its symbiosis with fundamental bacterial microbiome and viral components. It also detects for example aged self-cells and destroys them, and rejects transplanted elements from allogeneic individuals (Cohen and Efroni, 2019).

It is also important to note that the innate immune response is not solely the property of specialized immune cells. Epithelial cells, endothelial cells, and fibroblasts also express various types of pattern-recognition receptors that detect pathogen-associated molecular patterns (associated with foreign agents) and damage-associated molecular patterns (originating from the host’s own stressed, injured, or dying cells) (Yirmiya and Goshen, 2011; Franz and Kagan, 2017). Furthermore, numerous non-immune cell types are able to sense nucleic acids and secrete type-I interferons in response to foreign nucleic acids in their cytoplasm (Schlee and Hartmann, 2016). Therefore, non-immune cells participate in the early stages of the immune response by secreting antimicrobial peptides, pro-inflammatory cytokines, and chemokines (Gallo and Hooper, 2012; Turner et al., 2014), that alert, recruit and activate immune cells, thereby initiating the cascade of immune response.

Tissue-resident immune cells, along with some non-immune cells, implement tissue immune surveillance by continuously monitoring and receiving signals from their environment. When a threat is identified, tissue-resident immune cells attempt to resolve the issue. If the problem persists, the danger alarm spreads, leading to the recruitment of additional immune cells from distant areas of the tissue and vasculature. The escalation continues if the threat remains. The magnitude of the immune response might reach a point where it induces behavioral changes known as “sickness behavior”: anorexia, emotional disturbances, social withdrawal, anhedonia and cognitive impairment. Sickness behavior is triggered by systemic pro-inflammatory cytokines secreted by immune cells (Herz and Kipnis, 2016; Kipnis, 2016; Rankin and Artis, 2018). To prevent detrimental excessive immune responses, the immune network regulates itself to balance immune activation and suppression through a negative feedback loop (production of anti-inflammatory cytokines), specialized regulatory cells (suppressing immune response through direct cell-cell contact or secretion of anti-inflammatory cytokines), expression of immune checkpoint molecules on the immune cell surface, apoptosis, and other mechanisms (Opal and DePalo, 2000; Strasser et al., 2009; Josefowicz et al., 2012; Pardoll, 2012; Nagata and Tanaka, 2017).

An essential feature of the immune system is its ability to acquire memory–a key property of cognitive systems. This process occurs in both the innate and adaptive immune systems, leading to a more robust and rapid response upon re-exposure to a stimulus (Netea et al., 2011, 2020; Kurosaki et al., 2015). In addition to immune memory to antigen, adaptive immune cells undergo “training” during their development and maturation—a process called “selection.” During selection, cells that can recognize various antigens without exhibiting self-reactivity are chosen for survival and continue to mature, while those that do not meet these criteria are eliminated through apoptosis. The purpose of selection is to ensure that the adaptive immune system can mount an effective immune response against foreign antigens without harming its own tissues (Nemazee, 2006; Klein et al., 2014). The innate immune system also has examples of “training” for functional competence and self-tolerance (Jentho and Weis, 2021). For instance, natural killer (NK) cells, a population of innate immune cells, undergo a process called “NK licensing” or “NK cell education” during their development. During the licensing process, immature NK cells are tuned for responsiveness, resulting in the generation of licensed or unlicensed NK cells. Licensed NK cells are more functionally competent and responsive compared to unlicensed NK cells; however, both subpopulations are important parts of the immune system (Kim et al., 2005; Orr and Lanier, 2010; Tu et al., 2016).

The described characteristics of the immune network are aligned with cognitive processes such as perception, attention, decision-making, communication, problem-solving, learning, and memory. As previously mentioned, the immune system heavily communicates, interacts, and regulates (and is regulated by) other systems of the body (Eskandari et al., 2003; Hu and Pasare, 2013; Fleshner and Crane, 2017). The immune system, along with the neural and endocrine systems, are considered the major control systems in organisms, tightly linked to one another. Neurons create their own networks by connecting directly to each other via synapses, enabling rapid communication and quick information processing. In contrast, the endocrine and immune systems utilize the cardiovascular and lymphatic systems for distant communication. These three systems are closely connected, essentially forming a single network for information processing and action (Besedovsky and Rey, 2007; Dantzer et al., 2008; Dantzer, 2018). For instance, acute stress induced by physical and psychological conditions leads to the secretion of neurotransmitters and hormones such as corticotropin-releasing hormone, vasopressin, vasoactive intestinal polypeptide, serotonin, beta-endorphins, neuropeptide Y, adrenocorticotropic hormone, glucocorticosteroids, norepinephrine, and epinephrine (Black and Garbutt, 2002; Dhabhar et al., 2012; Weigent, 2013). Immune cells express receptors for glucocorticoids and catecholamines (α and β2-adrenoreceptors), enabling the immune network to perceive, pay attention to, and react to stress signals (Kohm and Sanders, 2000; Pavlov and Tracey, 2005; Drummond, 2014; Wohleb et al., 2015). In response to acute stress, some subsets of immune cells exit their depos into blood circulation and migrate to the barrier tissues, such as skin, in order to combat microorganisms in the event of skin damage (e.g., scratches or bites) (Schedlowski et al., 1993; Dimitrov et al., 2009; Dhabhar et al., 2012).

Interestingly, immune cells can be a source of peptide hormones and neurotransmitters such as acetylcholine, adrenocorticotropic hormone, endorphins, enkephalins, vasoactive intestinal peptide, substance P, vasopressin, atrial natriuretic peptide, and corticotropin-releasing hormone (Blalock, 2005; Blalock and Smith, 2007). Furthermore, cytokines produced by immune cells regulate neuronal function, influence brain development, and behavioral abnormalities (Yirmiya and Goshen, 2011; Bilbo and Schwarz, 2012; Choi et al., 2016; Kim et al., 2017). Moreover, the immune network can impact cognitive function through the modulation of pain (McMahon et al., 2005; Ren and Torres, 2009; Moriarty et al., 2011; Grace et al., 2014; Gupta and Harvima, 2018) or by involvement in the gut-brain axis (Sharon et al., 2016; Strandwitz, 2018).

An extensive and systematic review of the mechanisms enabling immune cells to dynamically wire their circuits and interactions throughout the body is outside the scope of this paper (see Rieckmann et al., 2017; Cohen and Efroni, 2019; Shilts et al., 2022 for a recent discussion). In what follows we focus on the brain-immune cellular networks relationship.

## 5. The brain-immune network

The immune system is uniquely like the brain: both brain and immune system develop fully, far beyond their genes, as a result of somatic lifetime experience (Cohen, 2000). Under the hierarchical perspective of the FEP, it has been argued that the brain and the immune system are internal states of the same Markov blanket and necessarily influence each other (Palacios et al., 2020; Bhat et al., 2021). Markov blankets are typically defined as a statistical boundary that separates two sets of states (e.g., a cellular membrane separating intracellular and extracellular dynamics) (Pearl, 1988; see Bruineberg et al., 2021 for a critical discussion).

Recent work by Schwartz et al. (2022) proposed the idea of a brain-immune network “ecosystem.” The received view considered indeed the brain as a self-contained tissue responsible for its own immune protection and equipped with microglia, acting as internal immune sentinels. However, as Shechter et al. (2013) note, the Central Nervous System (CNS) repair and higher brain function (Ziv et al., 2006) have been found to be dependent on adaptive and innate immune cells derived from the circulation. These findings opened the search for regions within the brain containing adaptive immune cells which are considered able to affect the brain *from distance*. Intriguingly, the discovery of border structures through which reparative immune cells can enter the brain to provide help without breaching the blood-brain barrier (Shechter et al., 2013) open a new window into the complex relationship between the CNS and the immune networks.

The brain-immune network “ecosystem” consists of the idea that “the cellular elements of this immunological network, together with the non-immune cells of the brain—neurons, astrocytes, and oligodendrocytes—constitute a functional structure with properties of an “ecosystem,” characterized by interdependent compartments of immune cells that interact with each other within a physically connected microenvironment, thereby contributing to increased stability and resilience of the CNS in the face of continuous disruption in its day-to- day activities.” [Schwartz et al. (2022):1].

It is also important to stress that the human brain is not composed of neurons only, but also of non-neuronal cell types. The latter may have a lower diversity than neurons in baseline adult state. Yet many non-neuronal cell types undergo significant changes, i.e., they exhibit many different cell states, under different physiological or diseased situations (Zeng, 2022). For example, astrocytes display complex morphological and physiological properties in different brain regions and contribute to essential functions in blood-brain barrier, synaptogenesis, neurotransmitter buffering, ion homeostasis, and secretion of neuroactive agents (Ben Haim and Rowitch, 2017).

Crucially, the cells called microglia are the primary innate immune cells in the central nervous system and have a distinct developmental origin from peripheral immune cells (Thion and Garel, 2020). They are generated from mesodermal progenitors that arise from the yolk sac and remarkably, they are among the earliest residential cell types in the brain. Microglia display diverse and dynamic phenotypic states and play a plethora of roles in development, adulthood (homeostasis), aging, and diseases (Butovsky and Weiner, 2018).

Based on the observation that immunity is not merely an automatic response to a foreign presence, it has been proposed to characterize immune processing as an “act of cognition” (Cohen, 1992). Since human bodies are biological *self-*organizing open systems, the development and function of a cellular network designed to keep track of the “self” in relation to both the external environment, and its internal structures may be seen indeed as an essential cognitive system. This system needs to be flexible and “smart” enough to decide on the fly whether certain elements are optimal for and/or belong or not to *this* self-organizing system. It also needs to compute whether certain self-organizing processes unfold according to the plan or go awry. Interestingly, this idea can be traced back to the seminal work of Varela and Coutinho (1991a),b; Varela et al. (1998) (see also Vaz, 2011) who paid careful attention to the special interplay between immune and somatic processes, coining the term “immunoknowledge” (Varela and Coutinho, 1991a).

One key observation is that taken in isolation, when confronted with a pathogen or incoming signal, each individual immune cell has a limited “view.” An individual cell is blind to information that does not directly activate its reception. To put it metaphorically: “each cell is confined to a world compressed by its own short-sightedness (Cohen and Efroni, 2019:3). Hence, in order to ensure flexible response to pathogens and other incoming signals, individual immune cells must coordinate and integrate their disparate responses to produce a systemic decision. Based on this observation, it has been proposed that the network of immune cells make “collective decisions through a type of self-organizing swarm intelligence or crowd wisdom “(Cohen and Efroni, 2019:1). This idea is in line with previous work showing that the collective coordinated behavior of cells composing an organ or a tissue requires information processing tracking the internal state of the neighboring cells (Perbal, 2003). It has been shown that the iterative exchange of information involving cell-to-cell communication may give rise to cooperative cellular behavior even under noisy conditions (Koseska et al., 2009).

In a similar vein and taking this line of reasoning one step further, one may argue that neuronal cells also, taken in isolation, may be equally blind to which type of information is noteworthy for optimal responsiveness and processing of key physiological states of the self-organizing biological system it supervises as a whole–the human body. The same way immune cells interact and communicate with each other to deliver flexible response to incoming signals, the neuronal cells need to interact not only with each other, but also with non-neuronal cells (e.g., immune cells) to coordinate joint responses. Collective teamwork of neuronal and immune signaling need to constantly orchestrate on the fly joint finetuned responses to meet constantly changing incoming signals from the body and the environment.

Moreover, not only individual cells, but also specialized organs and networks may equally have a limited “short-sighted” view of the world of the organism it composes and ensure self-preservation. Hence one may speculate that biological self-organization in the human body emerges as a “crowd wisdom” not only at the inter-cellular level subserving a given network (neural, immune, endocrinologic, etc.). Importantly, it emerges also at the inter-networks level, that is, from the interactions between different systems orchestrating their responses in tandem to address key challenges for the survival of the body. Given that biological organisms are fundamentally multi-scale evolving agents, cognitive processing should equally display a multi-scale distributed structure, with the immune system playing a central, yet overlooked role, working in tandem with the neural system (Varela and Coutinho, 1991a,b).

It is important to stress here that the aim here is not to provide an explanation to the perennial mind-body problem, i.e., how mental states emerge from physical states in the brain. The aim rather is to point out that the very distinction between mind and body, inherited from previous traditions, tacitly confines cognitive processing to the brain only. However, if cognition is defined as information processing, then all bodily cells are cognitive in this minimal sense, not just those operating in the brain.^2^

Up to now we have overviewed theoretical and empirical work suggesting that self-organizing biological systems such as human bodies are structured by complex sets of dynamically reciprocal pathways in which each neuron/cell both shapes and are shaped by the organism’s regulatory and developmental processes as a whole. In the last section, we take a closer look at the idea that cognitive processing is best described as an intricate interplay between both macro- and microscopic processing at hierarchical multisystem levels distributed across the whole organism.

Taken together, this robust body of evidence reviewed above points to the idea that cognition can be seen as a multiscale complex web of dynamic information processing distributed across multilevel cellular (e.g., neural and immune) and network systems operating across the entire body, and not just in the brain.

## 6. Brain-body multiscale distributed cognition

Prevailing approaches in cognitive neuroscience tacitly stipulate that brain states are somehow the natural, necessary home of cognitive information processing.

Yet, “smart” information processing seems to be pervasive at the non-neuronal cellular level as well. If this is so, then one may ask why cognitive and by extrapolation mental processes are so tenaciously associated with brain and neuronal processes only. This is an important and complex question that calls for careful and systematic consideration in future work. However, here we briefly list what we take to be some key elements supporting the tacit privilege of neurons over other type of cells in constituting cognition.

First, cognition has been examined preferentially from an adult-centric static approach, focusing on the brain as fully developed organ. However, it has been recently argued in favor of a developmental turn in understanding how brains and neurons emerge in relation to the rest of the other bodily organs and others’ bodies throughout the lifespan (Blakemore, 2012; Ciaunica and Fotopoulou, 2017; Ciaunica et al., 2021a,b). This dynamic approach may open new windows exploring how perception, cognition and self-processing temporally evolve from the womb into early infancy and beyond (Ciaunica and Crucianelli, 2019). Recent work has outlined that the boundaries between neuronal and non-neuronal cellular processing are much more complex and malleable than has been appreciated. This holds especially when one endorses a dynamic, developmental perspective on the growing brain and organism, tracing multi-layered cognition spanning from basic, cellular levels to higher psychological levels (Herrera-Rincon and Levin, 2018; Ciaunica et al., 2021a,b; Lyon et al., 2021).

For example, the transformation from unicellular to complex multicellular organisms requires the multiplication of the individual cells and the diversification of their function across the lifespan. The entire human repertoire of brain and body cell types are built, as Zeng notes, “through a sequential and parallel series of spatially and temporally coordinated developmental events starting from a single fertilized egg, the zygote” (Zeng, 2022:2748). The observation that cell type development is not a simple linear process but a highly multifaceted one invites us to reconsider the classical picture of a “pyramidal” hierarchical cell organization. Rather, a “tree of cell types” (Zeng, 2022: 2750) may be a more accurate picture for capturing the overarching classification of cell types and their complex relationships (Stadler et al., 2021).

Second, as Levin (2019) notes, traditional brain sciences operate with the built-in assumption that the body structure is tacitly taken to be fixed, determined by the genome and thus a reliable and stable machine for which appropriate control policies (i.e., behaviors) are implemented. Within this view, individual neurons coordinate to implement a higher-order entity, a “self” with coherent memories, beliefs, emotions, and plans. In short, brains are considered to be a stable, fixed structure in which the individuality of the immobile cells (very much like the bricks of a wall) disappears in the service of the adult body (despite the well-established turnover rate of neurons in adult human brains, which does not seem to alter self-continuity nor memory) (Spalding et al., 2013), and data on a variety of models in which memory appears to be not confined to brain tissues (Blackiston et al., 2015).

However, in healthy humans, successful survival of the organism cannot be done by neuronal processing alone, or in isolation of other key cellular processing. Rather, multiple cellular processing (e.g., immune system processing) must coordinate with the neural processing to achieve self-maintenance, and self-regulation of the biological system. For example, as illustrated earlier, immune and neural systems are intricate systems both composed from cells, communicating with each other, depending on each other and pursuing a common goal: self-preservation of the human body/organism (Varela and Coutinho, 1991a,b; Bhat et al., 2021).

Recently, several theorists joined voices to call for a reconceptualization of the fundamental basis of brain-body-behavior structural dynamics (Pessoa et al., 2021). For example, it has been argued that the vertebrate neuroarchitecture does not respect the boundaries of standard mental terms. Rather neuroscience should aim to address the dynamic coupling between large-scale brain circuits and complex, naturalistic behaviors” (Dennis et al., 2021; Pessoa et al., 2021; Branchi, 2022). These authors suggest that “brain evolution is better understood in terms of (i) modification in neuronal populations with the brain’s fundamental units (building blocks) and (ii) the reorganization of large-scale connectional systems in which they are engaged” (Pessoa et al., 2021:3; see also Pessoa et al., 2019).

Moreover, the neuroarchitecture is not additive, in the sense that new components are “added on atop an ancestral organization” (Pessoa et al., 2021:7). Rather, distributed brain circuits help solve challenging behavioral problems. Hence, standard mental properties (e.g., “decision-making”) are deeply intertwined with others (e.g., “affective processing”)” (Pessoa et al., 2021:7). The radical approach here is that standard mental categories such as perception, memory, perception and emotion may be ill-suited to investigating not only unconventional examples, such as slime mold memory (Vogel and Dussutour, 2016; Boussard et al., 2019), but also even the brain basis of behavior. This shift in focus invites neuroscience to consider the coupling between large-scale circuits and complex naturalistic behaviors by taking into account how the temporal evolution of behavior is linked to dynamic brain changes (Pessoa et al., 2021; Branchi, 2022).

Our proposal, although compatible with this approach, takes a step further and questions the very distinction between i) cognitive processes, supported by neural cells in the brain; ii) and bodily processes, supported by non-neural cells in the body. Rather, we suggest, all cells process information, make decisions, interact with each other, and as such, actively contribute to the survival of the biological organism as a whole.

This view echoes the enactive approach outlining that the interaction process itself constitutes an irreducible domain of dynamics which can be constitutive of individual agency and social cognition (De Jaegher and Froese, 2009). Importantly, it is possible to retrace the impact of such irreducible interactions between autonomous systems “all the way from cell to society” (Thompson, 2007; Levin and Dennett, 2020).

## 7. Conclusion and future prospects

This paper proposed a shift in perspective from neuronal to cellular (i.e., immune) processing as an essential step to understand the fundamental nature of human mental processes and cognition. This approach is in line with previous seminal embodied cognition views reframing cognition to reflect its fundamental biological organismic basis (Maturana and Varela, 1980; Lyon et al., 2021). The embodied and enactive approaches seminally claim that cognition (brain processes) necessarily requires interactions with the body and the environment to get off the ground.

Our suggestion is subtly yet importantly different: cognition is the result of information processing distributed across all cellular systems in the body, including the brain, which is, in our view, (part of) the body. Speaking about brain-body-environment interactions in constituting cognition may be misleading because it tacitly inherits the distinction between mind (brain) and body. Cognitive processing however, does not require brain *plus* body *plus* environment. Rather, cognitive processing takes place in every single cell of our bodies, among which neuronal cells play a key part, but only a part. Hence, one may say that we literally think with all the cells of our bodies, and not just our heads.

Our paper thus paper invites to a nuanced understanding of cognitive processing as cut across multiple levels of bodily systems and cellular processing (e.g., neuronal *and* immune). Cognition may be thus better understood as “multi-scale continuum of organizational levels of capabilities” (Levin, 2019) designed to subserve the self-organization and adaptation of the human organism as a whole, rather than a process restrictively confined to the brain and the neural system.

The ideas mentioned here may have the potential to open new avenues of investigation in several important ways. For example, as we mentioned earlier, it invites to reconsider the prevailing differentiation between the brain and the body as two distinct organismic categories. Rather, the brain *is* (part of) the body, and as such both neuronal and non-neuronal bodily cells and complex network systems should be explored as constitutive parts of one single self-organizing biological system, the human organism. Unless one endorses explicitly a brain (mind)-body dualistic stance–stipulating that the brain is fundamentally different from the rest of the other human organs, and hence, conceptually and ontologically separable from the body itself–the dichotomy between brain and body remains unwarranted from a purely neurobiological perspective.

Another interesting field in which the approach proposed here could have an impact is developmental cognitive neuroscience. The human brain is a critically a *developmental* system responding to perturbations in a manner that yields flexible yet robust behaviors, constantly adjusting to stressful and unforeseen conditions throughout the lifespan. For example, one important yet overlooked idea in current discussions in philosophy and cognitive neuroscience is that human brains and bodies first develop *within* another human body (Ciaunica, 2016; Ciaunica and Crucianelli, 2019; Ciaunica et al., 2021a,b).

Future work needs to address the fascinating bridge linking neural and immunological information processing occurring *between two* developing “co-embodied’ self-organizing systems, e.g., in pregnancy (Ciaunica et al., 2021b). Without a systematic investigation of the intricate, context sensitive processes that actively generate and shape the development of neurons and other bodily cells and systems throughout the lifespan in relation to others’ bodies, any efforts at present to understand the fundamental basis of human cognition will remain patchwork at best. Research focused on these flexibly co-emerging processes and developmental systems may provide substantial new causal insights into the nature of mental and cognitive processing in humans.

## Data availability statement

The original contributions presented in this study are included in the article/supplementary material, further inquiries can be directed to the corresponding authors.

## Author contributions

AC wrote the full draft. ML provided substantial feedback. ES provided additional substantial feedback and wrote the section on the immune system. All authors contributed to the article and approved the submitted version.

## Footnotes

1 We would like to thank an reviewer for pressing clarification on this point.

2 We would like to thank one reviewer for pressing clarification on this point.
